# Book Reviews

**Published:** 1896-09

**Authors:** 


Fifteenth Annual Report of the State Board of Health of New York. Including eighteen maps. Transmitted to the Legislature March 6, 1895. Albany: James B. Lyon, State Printer. 1895.

One of the most interesting portions of this report is that prepared by the commission on tuberculosis in cattle. It shows that great progress is making in this department of preventive medicine; for, whereas, prior to 1892 there was no law in existence in this state providing adequate authority to deal with the examination of cattle, there is now ample provision on the subject. It is asserted that tuberculosis causes one in every eight deaths in this state, which makes it of the first importance to throw safeguards around the milk supply. Milk is one of the principal aliments of childhood, and as the channel of infection is principally through the intestinal tract, the danger from the use of infected milk is very great.

During the first year and a half of the operation of the law the state board of health examined 22,000 cattle, of which number 800 were condemned and slaughtered. The methods of determining the presence of tuberculosis are now almost certain, and it is becoming that a great state like New York should exercise the most prudent care in its detection among herds and should institute the most summary measures to eradicate it when found. We commend the good work of the board in this direction and trust it will receive the support of all interested in the preservation of the public health. These volumes should be in possession of all sanitarians and public health officers.

---

INTERNATIONAL CLINICS. A Quarterly of Clinical Lectures on Medicine, Neurology, Surgery, Gynecology, Obstetrics, Ophthalmology, Laryngology, Pharyngology, Rhinology, Otology and Dermatology, and specially prepared articles on treatment. By professors and lecturers in the leading medical colleges of the United States, Germany, Austria, France, Great Britain and Canada. Edited by Judson Daland, M. D., (Univ. of Penna.) Philadelphia, Instructor in Clinical Medicine and Lecturer on Physical Diagnosis in the University of Pennsylvania; Assistant Physician to the Hospital of the University of Pennsylvania; Director of the Stetson Laboratory of Hygiene; Fellow of the College of Physicians of Philadelphia. J. Mitchell Bruce, M. D., F. R. C. P., London, England, Physician to, and Lecturer on, the Principles and Practice of Medicine at the Charing Cross Hospital. David W. Finlay, M. D., F. R. C. P., Aberdeen, Scotland, Professor of Practice of Medicine in the University of Aberdeen; Physician to, and Lecturer on, Clinical Medicine in the Aberdeen Royal Infirmary; Consulting Physician to the Royal Hospital for Diseases of the Chest, London. Volume IV. Fifth series. 1896. Octavo, pp. xii.—363. Philadelphia: J. B. Lippincott Co. 1896.

This volume, in continuation of an excellent series of clinical lectures, is to be characterised as one of the best that has been issued. John Ashurst contributes a lecture on tuberculosis of the hip-joint that contains much which is interesting if not new, and withal it is handsomely illustrated.

Mr. Christopher Heath presents a case of sarcomatous tumor of the upper jaws. With this he shows an excellent photograph of a case of epithelioma of both superior maxillæ. The picture is of great diagnostic significance.

Arpad G. Gerster lectures on the modern treatment of urethral stricture and the author's self-indicating bulbous aseptic urethrotome. He illustrates his instrument and also gives a plate showing paraffine casts of the normal urethra, taken from the cadaver, which exhibit the varying caliber of this canal in health. The importance of a better knowledge of these diseases is attracting the attention of genito-urinary surgeons in all parts of the world. Charles Cary lectures briefly on hemiplegia, and he is the only Buffalo representative in this volume. Perry H. Millard tells of spreading or progressive gangrene and makes an interesting and instructive lecture out of a very loathsome condition, which, however, is one of the most important in the whole range of surgical pathology. Joseph M. Mathews discourses upon syphilitic ulceration with stricture of the bowel ; also of combined internal and external hemorrhoids. Whenever Dr. Mathews speaks to his pupils he instructs them in a manner that they are not likely to forget. We should like to speak of several other lectures in this volume, did space permit.

---

Diagnosis and Treatment of Diseases of the Rectum, Anus and Contiguous Textures. Designed for Practitioners and Students. By S. G. Gant, M. D., Professor of Diseases of the Rectum and Anus, University and Woman's Medical Colleges; Lecturer on Intestinal Diseases in the Scarritt Training School for Nurses ; Rectal and Anal Surgeon to All-Saints, German, Scarritt's Hospital for Women, and Kansas City, Fort Scott and Memphis Railroad Hospitals, etc., etc. With two chapters on Cancer and Colotomy, by Herbert William Allingham, F. R. C. S., Eng., Surgeon to the Great Northern Hospital ; Assistant Surgeon to St. Mark's Hospital for Diseases of the Rectum ; Surgical Tutor to St. George's Hospital, etc., etc., London. One volume, royal octavo, 400 pages. Illustrated with sixteen full-page chromo-lithographic plates and 115 wood-engravings in the text. Philadelphia: The F. A. Davis Co., Publishers, 1914 and 1916 Cherry street ; New York: 117 W. Forty-second street ; Chicago : 9 Lakeside Building.

In presenting a notice of this book we wish we could feel that it was a necessity, or that it filled a complementary place in the literature of the subject of which it treats. Since Mathews's excellent work was published a few years ago, now carefully undergoing revision, there seems to have been very little room for another treatise on diseases of the rectum and we doubt if this book gains favor with the specialists in that important department of surgery.

In the first place the author's style is unfortunate ; perhaps some critic might characterise it with a harsher term. In treating of pruritus ani (page 237) the author says : "We have derived immediate relief from brushing the diseased parts with a solution of nitrate of silver, etc." This auto-clinical picture borders on the amusing.

In a general way it is proper to state that the methods of the author are such as meet the approval of specialists, but we are unable to determine the development of any new facts, or a recasting of any old ones, that are of sufficient importance to warrant the publication of a new treatise at the present time on diseases of the rectum and anus. Mathews's quarterly journal covers the field in this department most admirably and keeps specialists well advised of the progress making as rapidly as it occurs.

In omitting the diphthong the author's good judgment is to be commended. It is unpleasant in this day and age to see otherwise beautiful pages marred by a retention of this ancient and useless method of spelling. Some of the plates are excellent, but the novitiate would gain a very crude idea of the semi-prone posture from the illustration on page 21.

---

Transactions of the Seventeenth Annual Meeting of the American Laryngological Association, held in the City of Rochester, N. Y., June 17, 18 and 19, 1895. Edited by Charles H. Knight, M. D., Secretary. New York: D. Appleton & Co. 1896.

The president of this association for 1895, Dr. John O. Roe, of Rochester, very fittingly chose for the subject of his annual address, The relation of damp air to diseases of the air-passages, and considered the subject with special reference to the prevalence of these diseases in the lake region. It is well known that the climate of the region of the Great Lakes is especially productive of diseases of the nose, throat and lungs. We need not go far in a search for causes before we ascertain that the humidity and dampness of the atmosphere, associated with frequent, sudden and extreme variations in temperature, are adequate to solve the problem. Dr. Roe has dwelt upon this point with great clearness. He has also emphasised the importance of thorough drainage as a factor in mitigating the ill-effects of these atmospheric conditions. There are very many other papers in the book of absorbing interest to laryngologists, most of which possess the excellent merit of brevity. A paper by Mr. Charles H. Ward, the distinguished naturalist, on Nasal characteristics, human and comparative, possesses great interest and is well illustrated. This association is noted for the excellent character of its scientific work, and its annual volumes are eagerly sought by laryngologists in every part of the world.

---

Transactions of the American Association of Obstetricians and Gynecologists. Volume VIII. Eighth annual meeting, held at Chicago, September 24, 25 and 26, 1895. William Warren Potter, M. D., Secretary, Buffalo, N. Y. Philadelphia: Wm. J. Dornan, Printer, 100 N. Seventh street. 1890.

This association has again issued an interesting and valuable volume that will take an important place in the literature of obstetrics, gynecology, pelvic and abdominal surgery. The papers, as usual, are pointed and the discussions spirited. One of the excellent features of this association is that the Fellows do not waste words in fulsome praise of each other, but at once address themselves to the meat and kernel of the subject under debate, attacking its weak points, accentuating its strong ones and leaving its essential features so well analysed that the reader is not in doubt as to the bearing and weight that should be given to the author's views.

During the year four Fellows of the association have gone to their immortality—namely, Dr. Franklin Townsend, of Albany; Dr. J. Edwin Michael, of Baltimore; Dr. Thomas Keith, of London; and Dr. L. Ch. Boisliniere, of St. Louis. Appropriate memorials, accompanied by excellent portraits, are published of the deceased, written respectively by Drs. Albert Vander Veer, George H. Rohé, Leith Napier and Frank A. Glasgow. The book is well indexed and printed and handsomely bound.

---

An Inquiry into the Difficulties Encountered in the Reduction of Dislocations of the Hip. By Oscar H. Allis, M. D., Fellow of the College of Physicians and of the Academy of Surgery, Philadelphia, etc. The Samuel D. Gross Prize Essay. Philadelphia. 1896.

It is one of the conditions of this prize that the successful competitor shall publish his essay in book form, hence a few copies have been sent out to the medical journals. The author devotes about two-thirds of his treatise to what he is pleased to term an introductory study. In this the lesion is considered anatomically, geometrically and surgically. A careful analysis is given of all these divisions, and they are illustrated with schematic drawings that serve to make clear the most intricate parts of by far the most important dislocation that is likely to take place in the human body. The author then, in part second, considers the reduction of dislocations by manipulation, and gives a critical examination of the various methods proposed and the obstacles that beset each of them. Here again is displayed the author's talent for analysis with mathematical accuracy, and profuse schematic drawing are again made to illustrate his views.

This essay is the most classic contribution to the subject that has yet appeared.

---

Obstetric Accidents, Emergencies and Operations. By L. Ch. Boisliniere, A. M., M. D., LL.D., late Emeritus Professor of Obstetrics in the St. Louis Medical College; Honorary Fellow of the American Association of Obstetricians and Gynecologists, etc., etc. Duodecimo, pp. 381. Liberally illustrated. Price, $2.00 net. Philadelphia: W. B. Saunders, 925 Walnut street. 1896.

This book has been prepared by an experienced obstetrician and presents many important questions relating to obstetric accidents and emergencies with clearness and perspicacity. We are, however, unable to discover any new points of importance connected with those urgent obstetrical questions that tax the judgment and mental equipment of the physician. It cannot be regarded in any sense as an authoritative treatise, but will serve to arm the young practitioner with information that he must hurriedly seek when he has neither time nor opportunity to consult the standard works on obstetrics.

It is illustrated with very many excellent drawings and plates, the best of which have been taken from Dickinson. We are not quite able to see a great necessity for the presentation of this book to the profession at this time, but being here we are quite willing to state that it contains very many useful hints that are for the most part succinctly set forth in agreeable English.

---

A COMPEND OF GYNECOLOGY. By WILLIAM H. WELLS, M. D., Adjunct Professor of Obstetrics and Diseases of Infancy in the Philadelphia Polyclinic, etc. Quiz Compends, No. 7. Duodecimo, with 150 illustrations. Price, 80c. Philadelphia : P. Blakiston, Son & Co., 1012 Walnut street. 1896.

A COMPEND OF DISEASES OF CHILDREN. Especially adapted for the use of medical students. By MARCUS P. HATFIELD, A. M., M. D., Professor of Diseases of Children, N. W. U. Medical School, etc. Quiz Compends, No. 14. Duodecimo, second edition, thoroughly revised. Price, 80c. Philadelphia : P. Blakiston, Son & Co., 1012 Walnut street. 1896.

Since we must needs be inflicted with quiz compends it is best to have good ones such as these. Wells has grouped a great mass of facts into a very compact space, and speaking generally they are such as a student must acquire for his pass examinations. On page 21 he illustrates several postures by drawings made from photographs taken by the writer of this notice, though credit is not given to the original source.

Hatfield has also presented many important questions relating to pediatrics, that will prove useful to the undergraduate in refreshing his memory.

---

TRANSACTIONS OF THE MEDICAL SOCIETY OF THE STATE OF NEW YORK for the Year 1896. FREDERICK C. CURTIS, M. D., Secretary, Albany, N. Y. Philadelphia : Wm. J. Dornan, Printer, 100 N. Seventh street. 1896.

The annual volume of transactions put forth by this society is rarely disappointing. It usually issues the best book of transactions of any state medical society with which we are familiar. The present one is fully up to the high standard set by this society many years ago and has received favorable comment from several medical journals outside the boundary lines of its own jurisdiction. New York has, since 1806, exercised some form of control over the practice of medicine. It is now conceded on all hands that it has one of the best medical practice acts extant. This law was presented to the legislature by this society and was pushed to a conclusion mainly through its efforts over a formidable opposition. It has been now in operation long enough to make its workings effective in regard to the personnel of the state medical society and the quality of work that it turns out; hence, year by year, an improvement in its annual volume may be expected from a society that is full of progress—of tomorrows rather than of yesterdays.

---

Weekly Abstract of Sanitary Reports, issued by the Supervising Surgeon-General Marine Hospital Service. Volume X. 1895. Washington: Government Printing Office. 1896.

Though these reports come to hand weekly they are greatly enhanced in value by having them bound at the end of the year and sent out in this fashion. They contain a valuable amount of information on the subject of public health and hygiene, and are the only record that identifies the general government in any way with the question of public sanitation. None of the great powers is as indifferent to these matters as the United States—more's the pity. We hope to see the time when a department of health will be created, presided over by a cabinet officer, to be nominated by the American Public Health Association. This method of appointment would practically take the question out of politics, which we conceive to be quite important. Meanwhile, let the marine hospital service continue its good work, of which these annual volumes give abundant evidence.

---

Transactions of the Rhode Island Medical Society. Volume V. Part II. George D. Hersey, M. D., Secretary. Providence: Snow & Farnham. 1896.

This modest volume in paper cover records the proceedings of an old medical society. It consists of the transactions at four quarterly meetings, the annual address of the president, Dr. Geo. F. Keene, eight scientific papers, annual committee reports, obituary notices, and list of Fellows. The pages are numbered consecutive to Part I., and all parts may be bound together, when issued, forming one standard octavo volume. The total membership of the society is 243, which is an excellent showing for a state of its size.

---

Transactions of the American Dermatological Association, at its Nineteenth Annual Meeting, held at Montreal, Can., September 17, 18, 19, 1895. New York: Geo. L. Goodman. 1896.

Dermatology is of necessity one of the most distinct of the specialties. It is hardly possible for a general practiser of medicine to gain any success in the treatment of diseases of the skin.

The transactions of this association, therefore, chiefly possess interest for dermatologists. The association has been in existence about twenty years and has but thirty-eight members. It is probable that there are fewer physicians devoting themselves exclusively to the practice of dermatology than to any other specialty. This volume is well edited by the secretary, Dr. Charles Warrenne Allen, of New York, and it deserves a more permanent cover. Paper is not stable enough to endure the necessary wear and tear of a library book.

---

TENTH ANNUAL REPORT of the State Board of Health and Vital Statistics of the Commonwealth of Pennsylvania. Benjamin Lee, M. D., Secretary. Philadelphia: Clarence M. Busch, State Printer. 1895.

This is one of the most valuable health reports issued by any of the states. Its secretary is an experienced sanitarian, and the influence exerted by him is apparent throughout the volume. We can readily understand and appreciate the importance of retaining such a man as Dr. Lee in office year by year. It seems a pity that all the states could not do the same. The statutes pertaining to state boards of health should be so drawn as to prevent politicians from interfering with the administrative officers. When an experienced and capable sanitarian can be induced to take the office he should be kept indefinitely; when an intriguing politician obtains it he should be unceremoniously dismissed with the greatest promptitude.

---

KEIL'S MEDICAL, PHARMACEUTICAL AND DENTAL REGISTER-DIRECTORY AND INTELLIGENCER. With special Medical, Pharmaceutical and Dental Departments, containing detailed information of colleges, hospitals, asylums, societies; with street lists, etc., etc., for Pennsylvania, New York, New Jersey, Maryland, Delaware and District of Columbia. Fourth edition. GEORGE KEIL, editor. Philadelphia: Burk & McFetridge Co., Publishers, 306 Chestnut street. 1896.

The editor has succeeded in grouping a vast amount of information, in easily accessible form, between the covers of this convenient volume. He has taken pains to perfect the list of medical societies, dispensaries and other organisations, and it may be considered altogether as the most complete and convenient medical directory that is published within the limits of its own boundary lines.

---

PROCEEDINGS OF THE PHILADELPHIA COUNTY MEDICAL SOCIETY. ALFRED STENGEL, M. D., Editor. Volume XVI. Session of 1895. Philadelphia: William J. Dornan, Printer. 1895.

We know of no county society that can compete with this one, either in the quality of the scientific work it does or in the method of sending it out to the profession. Its papers are carefully prepared and its discussions well reported. At the end of a year they are substantially bound into book form, with durable cover and gilt tops. The president during 1895 was Dr. James C. Wilson, under whose administration this book of 400 pages was created. The editor has performed his part admirably, and the printer has dressed the book in proper garb. Many of the papers possess scientific qualities of the first order.

---

Transactions of the Medical Association of Central New York. Twenty-eighth annual meeting, held at Syracuse, N. Y., October 15, 1895. William C. Krauss, M. D., Editor. Volume II. Buffalo: Buffalo Medical Journal Print. 1896.

That this association has adopted a good method for the preservation of its work, this second volume bears additional testimony to, and indicates an increased prosperity. The papers herein published, together with the discussions, have already been printed in the Journal, hence our readers have had an opportunity to become informed as to their literary and scientific worth. The volume is illustrated with a number of half-tone pictures, and is handsomely printed with clear type on good paper. It is a record of creditable work by an experienced association.

---

Twenty-first Annual Report of the State Board of Health of Michigan, for the year ending June 30, 1893. Henry B. Baker, M. D., Secretary. Lansing: Robert Smith & Co., State Printers. 1896.

Michigan is one of the foremost states in its sanitary administration. The secretary of the Michigan state board of health is an expert in sanitary science and is retained in office year after year, no matter what other changes take place in reference to its state board of health. This is a correctly set pace and one that New York could well afford to follow. Until it does it will never take its appropriate place among the most advanced commonwealths in regard to the prevention of disease. The annual volumes of the Michigan board are full of interesting material, this one being no exception to the rule.

